# Assessing the Predictive Power of the Hemoglobin/Red Cell Distribution Width Ratio in Cancer: A Systematic Review and Future Directions

**DOI:** 10.3390/medicina59122124

**Published:** 2023-12-05

**Authors:** Donatella Coradduzza, Serenella Medici, Carla Chessa, Angelo Zinellu, Massimo Madonia, Andrea Angius, Ciriaco Carru, Maria Rosaria De Miglio

**Affiliations:** 1 Department of Biomedical Sciences, University of Sassari, 07100 Sassari, Italy; dcoradduzza@uniss.it (D.C.); azinellu@uniss.it (A.Z.); 2 Department of Chemical, Physical, Mathematical and Natural Sciences, University of Sassari, 07100 Sassari, Italy; sere@uniss.it; 3 Department of Medicine, Surgery and Pharmacy, University of Sassari, 07100 Sassari, Italy; c.chessa17@studenti.uniss.it (C.C.); madonia@uniss.it (M.M.); 4 Istituto di Ricerca Genetica e Biomedica (IRGB), Consiglio Nazionale delle Ricerche (CNR), Cittadella Universitaria di Cagliari, 09042 Cagliari, Italy; andrea.angius@irgb.cnr.it; 5 Control Quality Unit, Azienda-Ospedaliera Universitaria (AOU), 07100 Sassari, Italy

**Keywords:** hemoglobin to red cell distribution width, Hb/RDW ratio, cancer, risk assessment

## Abstract

*Background and Objectives*: The hemoglobin (Hb)/red cell distribution width (RDW) ratio has emerged as an accessible, repeatable, and inexpensive prognostic factor that may predict survival in cancer patients. The focus of this systematic review is to investigate the prognostic role of the Hb/RDW ratio in cancer and the implications for clinical practice. *Materials and Methods*: A literature search of PubMed, Scopus, and Web of Science databases was performed by an independent author between 18 March and 30 March 2023 to collect relevant literature that assessed the prognostic value of the Hb/RDW ratio in cancer. Overall survival (OS), progression-free survival (PFS), and the association of these with the Hb/RDW ratio were considered to be the main endpoints. *Results*: Thirteen retrospective studies, including 3818 cancer patients, were identified and involved in this review. It was observed that, when patients with a high vs. low Hb/RDW ratio were compared, those with a lower Hb/RDW ratio had significantly poorer outcomes (*p* < 0.05). In lung cancer patients, a one-unit increase in the Hb/RDW ratio reduces mortality by 1.6 times, whilst in esophageal squamous-cell carcinoma patients, a lower Hb/RDW ratio results in a 1.416-times greater risk of mortality. *Conclusions*: A low Hb/RDW ratio was associated with poor OS and disease progression in patients with cancer. This blood parameter should be considered a standard biomarker in clinical practice for predicting OS and PFS in cancer patients. Future searches will be necessary to determine and standardize the Hb/RDW cut-off value and to assess whether the Hb/RDW ratio is optimal as an independent prognostic factor or if it requires incorporation into risk assessment models for predicting outcomes in cancer patients.

## 1. Introduction

Cancer poses a significant global health challenge, accounting for substantial morbidity and mortality, with 19.3 million new cases and 10 million deaths reported worldwide in 2020 [1]. There is a high prevalence of cancers such as female breast cancer, lung cancer, and colorectal cancer, influenced by factors like lifestyle, environment, and screening practices. Notably, breast cancer has become the most-diagnosed cancer globally, and thyroid cancer rates have surged due to overdiagnosis [2,3]. While developed countries exhibit higher cancer incidence rates, mortality rates vary less. Globally, prostate cancer is frequently diagnosed, with the highest rates in specific regions, but mortality rates for breast and cervical cancers are notably higher in developing countries due to lifestyle, environmental, and healthcare disparities [4,5,6,7,8,9]. The global cancer burden is projected to increase by 47% from 2020 to 2040, with a more substantial increase in developing countries [10]. This growth is fueled by demographic changes and increasing risk factors associated with globalization and economic growth [11]. Addressing this challenge necessitates the establishment of sustainable infrastructure for cancer prevention and care in developing countries [12]. Tailored interventions can reduce the future cancer burden and bridge disparities between developing and developed nations [13]. Public health policies should prioritize building such infrastructure to mitigate the growing cancer challenge [12].

In this context, the development of novel tools is critical and must emphasize clinical applications and translational research [14,15,16].

An augmented red cell distribution width (RDW) serves as a crucial measure, indicating the variability in red blood cell (RBC) size. The RDW, expressed as a percentage in the complete blood count, aids in the diagnosis and monitoring of various medical conditions [17].

An elevated RDW often indicates a mix of large and small red blood cells, signifying diverse potential causes for anemia. Scientific interest in the clinical utility of the RDW has been observed in specific disease states, such as obstructive sleep apnea syndrome (OSAS), chronic obstructive pulmonary disease (COPD), immune disorders, surgical procedures, retinal artery occlusion, and even COVID-19 [18,19,20].

The clinical relevance of the RDW extends beyond anemia, demonstrating significance for various disease states, including OSAS, COPD, immune disorders, surgical procedures, retinal artery occlusion, and even COVID-19 [21]. Conditions such as iron, vitamin B12, and folate deficiencies contribute to alterations in the RDW by impacting the production and size of RBCs [22]. Additionally, hemoglobinopathies like thalassemia and chronic inflammatory conditions such as rheumatoid arthritis and lupus are associated with an elevated RDW [21,23]. Diseases affecting the bone marrow, including myelodysplastic syndromes and leukemia, further influence the production of RBCs, which is reflected in the RDW. Moreover, the RDW emerges as a potential biomarker in cardiovascular diseases, linking an increased RDW to a higher risk of adverse cardiovascular events [24]. It is crucial to emphasize that while the RDW is valuable, its interpretation is most effective when considered in conjunction with other clinical and laboratory parameters [9,25,26,27,28,29,30,31]. This comprehensive evaluation is vital for informed clinical decision making. Its proposed role as a biomarker extends to certain cancers, potentially indicating larger tumors and advanced cancer stages.

Similarly, hemoglobin (Hb) levels have historically been indicators of a patient’s tolerance to treatment. Changes in Hb levels can influence decisions about the intensity and duration of therapy. Low Hb levels may indicate malnutrition, such as anemia, which potentially indicates a low tolerance to treatment [24,25]. Substantial evidence suggests that anemia before treatment may predict poor outcomes in cancer patients, including nasopharyngeal carcinoma, head and neck cancer, cervical cancer [26,27,28,29,30], and colorectal cancer [32]. Hb levels play a crucial role in diagnosing and monitoring various medical conditions. In anemia diagnosis, Hb levels are a primary indicator, suggesting the reduced blood oxygen-carrying capacity that is characteristic of various types of anemia [29,30]. Hb levels can reflect nutritional deficiencies, especially iron-deficiency anemia. Insufficient iron intake can lead to decreased Hb production, affecting overall health [28].

Hb levels are monitored to assess blood loss, either due to acute events like trauma or due to chronic conditions such as gastrointestinal bleeding. Certain chronic diseases, such as chronic kidney disease, can impact Hb levels, necessitating monitoring for effective management [29]. Hb is essential for transporting oxygen throughout the body, and abnormal Hb levels may indicate issues with oxygen delivery, affecting overall tissue and organ function. Various blood disorders, including sickle cell anemia and thalassemia, which are characterized by an abnormal Hb structure or production, can be diagnosed and managed through monitoring Hb levels [30]. Hb is linked to cardiovascular health, and abnormal levels may be associated with conditions such as heart failure and COPD [33]. In pregnant women, monitoring Hb levels is essential to detect and manage conditions like iron-deficiency anemia, which impacts both maternal and fetal health [34]. Hb levels are monitored postoperatively to assess blood loss during surgery and to guide transfusion decisions if necessary [35]. In summary, Hb levels are a versatile marker used in the diagnosis and the monitoring of various medical conditions, providing valuable insights into overall health, nutritional status, blood disorders, and treatment tolerance [36].

The relationship between the RDW and Hb levels in the context of cancer is intricately influenced by various non-neoplastic factors. These include deficiencies in essential nutrients, such as iron, vitamin B12, and folate [37]. Additionally, chronic inflammatory conditions, such as rheumatoid arthritis, systemic lupus erythematosus, and inflammatory bowel diseases, can exert an impact on both the RDW and Hb levels [38]. Certain chronic diseases, including chronic kidney disease and COPD, contribute to alterations in the RDW and Hb. Conditions leading to the increased destruction of red blood cells (hemolysis) can significantly influence both the RDW and Hb levels. Moreover, impaired kidney function has repercussions on erythropoiesis, manifesting as changes in the RDW and Hb. Diseases affecting the bone marrow, such as myelodysplastic syndromes and leukemia, play a role in shaping the production and maturation of red blood cells, thereby affecting the RDW and Hb [39]. Furthermore, specific drugs and medications may impact the production and survival of RBCs, exerting an influence on the RDW and Hb [40]. Inherited conditions, like thalassemia and sickle cell anemia, contribute to variations in both the RDW and Hb [41]. Disorders influencing the structure or synthesis of hemoglobin, such as thalassemia, can similarly impact both the RDW and Hb.

In the realm of cancer research, the Hb/RDW ratio emerges as a pivotal biomarker that was initially proposed as a prognostic indicator in esophageal squamous-cell carcinoma (ESCC) [23] and subsequently applied across diverse cancer types [34,42,43]. Demonstrating its utility as an independent prognostic factor, the Hb/RDW ratio significantly influences both overall survival (OS) and disease-free survival.

This ratio provides valuable insights into RBCs’ size distribution and oxygen-carrying capacity, which are essential aspects influenced by cancer-related factors. As cancer often induces alterations in blood parameters, the Hb/RDW ratio gains particular significance [43,44]. Changes in the RDW and Hb levels, influenced by factors like inflammation, nutritional deficiencies, or bone marrow disorders, directly impact the Hb/RDW ratio. Monitoring this ratio over time becomes crucial for gaining valuable information on cancer progression and prognosis, establishing itself as a promising tool in cancer prognosis research. The Hb/RDW ratio acts as a composite measure, offering a comprehensive perspective on RBCs’ distribution and oxygen-carrying capacity, which are both critical elements that are affected by various cancer-related factors.

The Hb/RDW ratio has been investigated in various cancer types, demonstrating its correlation with tumor characteristics and progression. In hepatocellular carcinoma (HCC), the Hb/RDW ratio was identified as a significant factor influencing progression-free survival (PFS) and OS [45]. Studies in hematologic cancers revealed a significantly higher Hb/RDW ratio in patients with endometrial carcinoma and primary HCC, which was associated with poor survival outcomes [46,47].

Cancer-related physiological changes play a substantial role in influencing the Hb/RDW ratio [48]. The systemic nature of cancer induces physiological stress, triggering changes in blood parameters such as the RDW and Hb, subsequently affecting the Hb/RDW ratio [49]. Chronic inflammation, a common response to cancer, impacts both the RDW and Hb levels, contributing to alterations in the Hb/RDW ratio [50]. Conditions often associated with cancer, such as deficiencies in iron, vitamin B12, and folate, can impact the production and size of RBCs, further affecting the Hb/RDW ratio [17]. Tumors affecting the bone marrow, as seen in myelodysplastic syndromes and leukemia, can disrupt the normal production of RBCs, manifesting as changes in the RDW and Hb levels, ultimately influencing the Hb/RDW ratio [51].

Furthermore, advanced tumor stages and larger tumor sizes have been linked to changes in the RDW, offering insights into tumor characteristics and progression [52]. Therapies like chemotherapy and radiation, which directly affect bone marrow and the production of RBCs, contribute to the composite nature of the Hb/RDW ratio, making it a valuable indicator of a patient’s overall condition [53,54].

**Figure 1:**
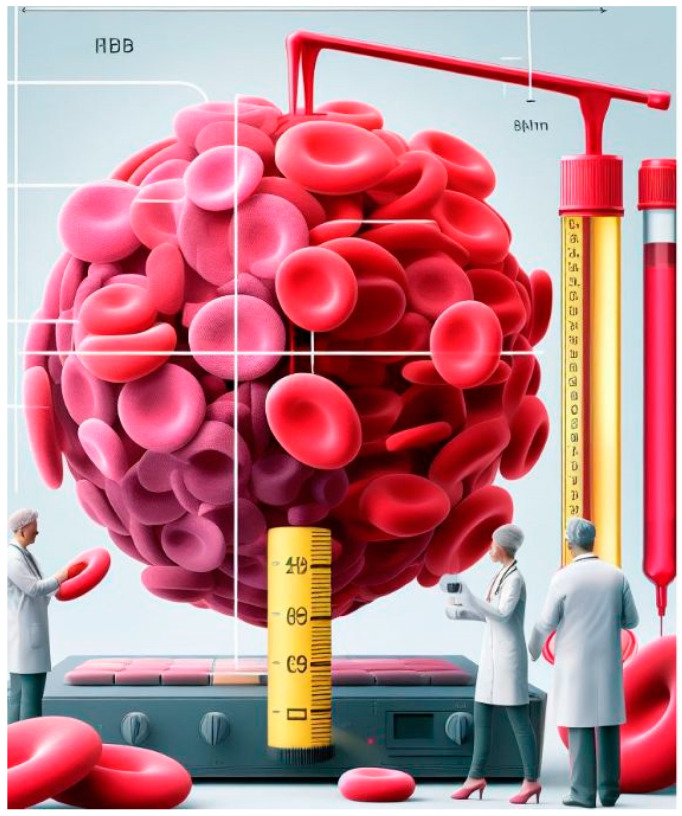
Critical role of hemoglobin (Hb)/Red Cell Distribution Width (RDW) ratios in utilizing blood cell data for diagnostic and prognostic purposes. Hb levels and RDW are fundamental parameters analyzed from blood samples, and their ratio (Hb/RDW) serves as a valuable metric in clinical assessment.

This systematic review aims to comprehensively explore the prognostic value of the Hb/RDW ratio in cancer, shedding light on its implications for clinical practice. Figure 1 provides a visual representation of the key components and relationships discussed in this review.

## 2. Materials and Methods

### 2.1. Search Strategy and Selection Criteria

This systematic review adheres to the PRISMA guidelines and aligns with the Grades of Recommendation, Assessment, Development, and Evaluation (GRADE) criteria [45,47,55]. From 18 March to 30 March 2023, the authors conducted a comprehensive search on the PubMed, Scopus, and Web of Science databases. Utilizing keywords such as “Hb/RDW”, “hemoglobin to red cell distribution width”, “HRR”, “hemoglobin”, “red cell distribution width”, “cancer”, “prognosis”, “prognostic value”, “overall survival”, “progression-free survival”, and “event-free survival”, the search aimed to refine the scope of the literature.

Inclusion criteria involved the exploration and reporting of the Hb/RDW ratio’s value in cancer patients. Literature was excluded if it fell into categories such as review articles, pre-2013 publications, or non-English language articles. Additional relevant material was sought by screening the reference lists of the identified literature and previous review articles.

The exclusion criteria were carefully applied, encompassing materials like abstracts, letters, reviews, case reports, etc. Studies with insufficient data for comprehensive analysis were omitted. Research lacking specific data regarding hematologic malignancies or the RDW was excluded. In cases where multiple publications originated from the same cohort, only the most recent report was included for our meta-analysis.

### 2.2. Data Collection and Quality Assessment

The literature satisfying our eligibility criteria and incorporated into this review underwent data extraction by the authors. The key data points comprised details of the study (author and date), the study design, the total number of patients, their cancer diagnoses, the specific outcomes measured, and the study results. The assessment of the literature incorporated in this review aligned with the GRADE criteria, which assesses the quality of evidence and provides recommendations for use [45]. These criteria encompass the quality of the methodology, the directness of evidence, heterogeneity, the precision of effect estimates, and the potential for publication bias. This resulted in assigning a level of evidence and recommendation for use, categorized as high, moderate, or low.

## 3. Results

### 3.1. Literature Search and Study Characteristics

**Figure 2:**
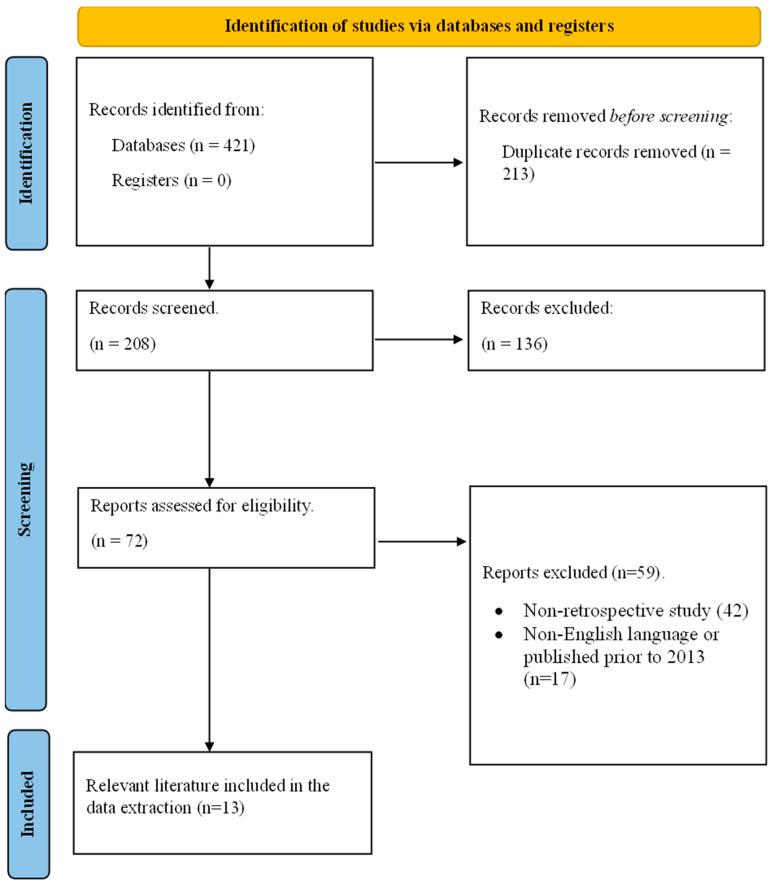
PRISMA flow diagram representing the literature search and study selection process.

The search revealed 421 papers, with 213 duplicates among them. Following the assessment of titles and abstracts from the remaining 208 papers, 72 full texts underwent review. In total, 13 studies met the specified criteria, and no further literature was identified in the references of the incorporated studies or by the reviewer team (Figure 2). All studies integrated in this systematic review and meta-analysis followed a retrospective design, in alignment with the inclusion criteria depicted in the methodology. The number of patients investigated ranged from 80 to 840, with a total of 3818 patients included across all 13 studies. The value of the Hb/RDW ratio as a prognostic measure was evaluated in lung cancer, upper tract urothelial carcinoma, esophageal carcinoma, renal cell cancer, bladder cancer, gastric carcinoma, lymphoma, head and neck cancer, breast cancer, and nasopharyngeal cancer. The comprehensive data extraction is described in Table 1.

### 3.2. Prognostic Value of Hb/RDW

All included literature reported a significant association between the Hb/RDW and prognostic outcomes of patients with cancer, including OS, PFS, and EFS (*p* < 0.05 to *p* < 0.001). In patients with small-cell lung cancer, a one-unit increase in the Hb/RDW ratio reduced death and increased survival by 1.6 times and had a statistically significant effect on OS and PFS [69,70]. Similarly, in patients with esophageal cancer, a lower Hb/RDW ratio was associated with a 1.416-times greater risk of mortality through the follow-up [71]. These associations were observed across all included cancer types aside from head and neck carcinoma. However, Tham et al. reported that the Hb/RDW ratio was independently related to event-free survival (EFS, *p* = 0.017) [42,43,72,73,74,75].

### 3.3. Other Outcomes

Beyond the association of the Hb/RDW ratio with primary parameters of survival, the identified literature also reported the prognostic value of the Hb/RDW ratio for predicting renal function and pathological staging in upper tract urothelial carcinoma patients. In these patients, a Hb/RDW ratio below 1.05 was related to a poorer renal function, and a tumor with a high pathological stage and grade [76]. It was observed in one study that a low Hb/RDW was linked with prolonged hospitalization, a higher RDW, and lower Hb levels (*p* < 0.05) [53,77,78,79].

### 3.4. Risk of Bias and Quality of Evidence

The assessment of the included studies’ risk of bias and the quality of evidence has been crucial for interpreting the findings and making informed conclusions. In this systematic review, the retrospective nature of the included literature presents inherent limitations in the study design, primarily in terms of selection and recall biases, as described by Talari et al. [80], Table 2.

Risk of Bias:

All the studies included in this systematic review followed a retrospective design, aligning with the predefined inclusion criteria. However, retrospective studies inherently carry risks of bias, particularly in terms of patient selection, data collection, and potential recall biases. The absence of a prospective approach limits the ability to control for confounding variables and may impact the robustness of the findings. Moreover, the inclusion of only retrospective studies may introduce a selection bias, as certain relevant data from prospective studies might have been excluded.

Quality of Evidence:

The GRADE evaluation encompasses considerations such as the methodological quality, the directness of the evidence, heterogeneity, the precision of effect estimates, and the risk of publication bias. All studies included in the review followed a retrospective design, but, as per the GRADE criteria, retrospective studies are considered lower in methodological quality compared to prospective ones. This limitation is acknowledged in the risk-of-bias assessment. The evidence directly addresses the prognostic value of the Hb/RDW ratio in various cancers, aligning with the research question. However, the retrospective nature of the studies may have impacted the directness of the evidence due to potential biases and confounding factors. Moreover, the included studies cover a diverse range of cancer types, which may contribute to heterogeneity in the findings. Heterogeneity can affect the generalizability of the results and should be considered in the interpretation.

The precision of effect estimates relies on the consistency and accuracy of the data. The retrospective design and potential biases may have impacted the precision of the effect estimates, and this limitation is reflected in the risk-of-bias assessment.

## 4. Discussion

This systematic review delves into the prognostic significance of the Hb/RDW ratio in cancer, aiming to shed light on its potential implications for clinical practice. The Hb/RDW ratio emerges from the literature and our findings as an accessible, repeatable, and cost-effective prognostic factor that is capable of predicting survival across various cancer types [23,29,81].

Our results consolidate evidence demonstrating the substantial prognostic value of the Hb/RDW ratio in lung, breast, gastric, esophageal, and lymphoma cancers [82]. The overarching trend establishes a correlation between a low Hb/RDW ratio and poorer OS, PFS, and EFS compared to cases with a higher ratio. This association is not only consistent but also notably significant in specific cancer subtypes, such as ESCC and pulmonary large-cell neuroendocrine carcinoma [16,30].

Moreover, the Hb/RDW ratio demonstrates its predictive capabilities post-treatment, as observed in gastric cancer patients treated with neo-adjuvant fluorouracil, leucovorin, oxaliplatin, and docetaxel (FLOT) [32]. These findings advocate for the broad clinical applications of the Hb/RDW ratio in cancer, warranting further exploration of its potential as a reliable prognostic marker.

In the era of advanced technologies like genomics, proteomics, and imaging, the financial implications of biomarker screening programs are well-documented [83]. Our study underscores the cost-effectiveness of the Hb/RDW ratio, positioning it as a practical and valuable screening tool for cancer. The observations of Toumazis et al. on the limited cost-effectiveness of biomarkers costing USD 750 or more highlight the pragmatic appeal of the Hb/RDW ratio, easily obtained from routine complete blood cell counts [41,42].

While the primary focus of this systematic review is the prognostic value of the Hb/RDW ratio, its diagnostic potential in cancer patients is noteworthy. The findings of Lin et al. on its utility in the auxiliary diagnosis of nasopharyngeal cancer, especially when combined with other ratios like the neutrophil-to-lymphocyte ratio or the platelet-to-lymphocyte ratio (PLR), extend its clinical relevance beyond prognosis [37]. Zhai et al.’s proposed combination of the Hb/RDW ratio with platelet/lymphocyte ratios (HP + PLR) further validates its efficacy as a simple and reliable prognostic marker, surpassing alternative indicators [43,44].

However, it is imperative to consider certain exclusions for the Hb/RDW ratio to maintain its prognostic value. Chronic inflammatory and autoimmune diseases must be ruled out, as emphasized by Su et al. [27]. Additional factors, including white blood cell count and platelet count, should be factored into the assessment, particularly in upper tract urothelial cancer patients.

Transitioning to the physiological underpinnings, the Hb/RDW ratio becomes a dynamic parameter reflecting changes during cancer development. The increased production of red blood cells, stimulated by tumor-induced metabolic demands and cytokines/growth factors, offers mechanistic insights. The nuanced relationship between cancer cells’ higher rate of cell division, the cytokine-induced production of RBCs, and the body’s response to cancer-induced anemia contributes to the variability in the Hb/RDW ratio.

The co-analysis of Hb and the RDW underscores their roles as crucial physiological markers during cancer progression and treatment. Hemoglobin’s susceptibility to decreasing due to chemotherapy-induced anemia and due to direct effects on the bone marrow positions it as a prognostic marker, especially in head and neck cancer. The association of the RDW with adverse prognostic factors in hematological lymphoma signifies its potential inclusion in prognostic scores for HL, given its simplicity, affordability, and easy availability [84].

In conclusion, the Hb/RDW ratio emerges as a promising, cost-effective, and easily accessible prognostic and diagnostic tool in cancer. Its versatility across cancer types, coupled with its mechanistic insights into physiological changes, warrants further exploration and validation in prospective studies. The limitations, particularly the retrospective nature of the included literature, advocate for future large-scale, multicenter prospective studies to standardize the Hb/RDW cut-off values and solidify its prognostic value.

## 5. Conclusions

In conclusion, this systematic review explores the prognostic significance of the Hb/RDW ratio in various cancers, shedding light on its potential implications for clinical practice. The global cancer burden is on the rise, necessitating innovative approaches to diagnosis, monitoring, and prognosis. As established in the introduction, cancer is a complex and multifaceted challenge influenced by various factors, including lifestyle, environment, and screening practices.

The review emphasizes the critical role of Hb and the RDW in cancer prognosis, with a focus on their dynamic interplay in the Hb/RDW ratio. An elevated RDW and altered Hb levels can serve as indicators of diverse medical conditions, ranging from nutritional deficiencies to chronic diseases and cancers. The systematic literature search and the meta-analysis, conducted in accordance with established guidelines, reveal a significant association between the Hb/RDW ratio and prognostic outcomes in various cancer types.

The findings underscore the versatility of the Hb/RDW ratio as a prognostic marker, demonstrating its value in predicting OS, PFS, and EFS. Notably, the association holds across different cancer subtypes, indicating the potential broad clinical applicability of this ratio.

Moreover, this review addresses the cost-effectiveness of the Hb/RDW ratio as a screening tool, contrasting it with more expensive biomarkers. The simplicity and the accessibility of the Hb/RDW ratio, derived from routine complete blood cell counts, position it as a practical and valuable option for cancer prognosis.

While focusing primarily on prognosis, this review also acknowledges the diagnostic potential of the Hb/RDW ratio, especially when combined with other ratios or parameters. The physiological underpinnings of the ratio are explored, highlighting its dynamic nature reflecting changes during cancer development.

Despite the compelling findings, this review acknowledges the limitations inherent in the retrospective nature of the included studies. To address this, we call for future large-scale, multicenter prospective studies, aiming to standardize the Hb/RDW cut-off values and to solidify its prognostic value across different cancer contexts.

In summary, the Hb/RDW ratio emerges from this review as a promising, cost-effective, and easily accessible prognostic and diagnostic tool in cancer. Its versatility, coupled with mechanistic insights into physiological changes, warrants further exploration and validation in prospective studies, offering a potential breakthrough in the landscape of cancer prognosis and patient management.

## Figures and Tables

**Table 1:** Data extraction and baseline characteristics of the identified literature.

Reference	Author (Year)	Study Design	Patient Population	Outcome Measures	Results
[56]	Su et al. (2021)	Retrospective	730 patients with upper tract urothelial carcinoma	Clinicopathological measures compared with Hb/RDW ratio	Patients with an Hb/RDW ratio below 1.05 showed a poorer renal function, tumor with high pathological stage, and high grade.
[57]	Figen et al. (2023)	Retrospective	840 patients with small-cell lung cancer	OS PFS RDW and Hb and associated ratios	A one-unit increase in Hb/RDW ratio reduced mortality and increased survival by 1.6 times.
[58]	Petrella et al. (2021)	Retrospective	342 patients with lung adenocarcinoma operated in the last two years	Preoperative Hb/RDW, Pathological stage Disease characteristics	DFS had an increased HR of relapse for preoperative Hb/RDW ratio lower than 1.01 (*p* < 0.004).
[59]	Sun et al. (2016)	Retrospective	362 patients ESCC patients	Hb/RDW ratio, OS, 5-year OS	Patients with a lower Hb/RDW ratio showed a 1.416 times greater risk of dying through the follow-up compared to healthy patients.
[60]	Yilmaz et al. (2021)	Retrospective	198 patients with RCC	Hb/RDW ratio, systemic immune-inflammation index, LMR, NLR, OS, PFS	Hb/RDW ratio is an independent prognostic factor for predicting PFS and OS in RCC patients.
[61]	Zhao et al. (2022)	Retrospective	80 patients with pulmonary large-cell neuroendocrine carcinoma	Hb/RDW ratio, characteristics, risk factors for OS	Patients with low Hb/RDW ratio exhibited a poorer OS than those with a high ratio (*p* < 0.001).
[62]	Yilmaz et al. (2020)	Retrospective	152 patients with muscle-invasive bladder cancer	Hb/RDW ratio, systemic immune-inflammation index, LMR, NLR, OS, PFS	Hb/RDW ratio is an independent prognostic factor for PFS and OS in patients with muscle-invasive bladder cancer
[63]	Yilmaz et al. (2020)	Retrospective	85 patients with gastric cancer who were treated with neoadjuvant FLOT	Hb/RDW ratio, DFS, PFS, NLR, systemic immune-inflammation index	Hb/RDW ratio was an independent prognostic factor for DFS and OS (*p* = 0.001 and *p* = 0.037, respectively); higher Hb/RDW was associated with better DFS and OS in gastric cancer.
[64]	Dong et al. (2022)	Retrospective	265 patients with DLBCL	Hb/RDW ratio, OS, PFS	Hb/RDW ratio is an independent prognostic factor for OS (*p* < 0.001) and PFS (*p* < 0.001) in DLBCL patients.
[65]	Tham et al. (2018)	Retrospective	205 patients with head and neck cancer	Hb/RDW ratio, EFS, OS	Multivariate analysis identified as independent prognostic factors associated with EFS: BMI (*p* = 0.0364), advanced T stage (*p* = 0.001), and low Hb/RDW ratio (*p* = 0.017). Hb/RDW was not associated with OS.
[66]	Bozkaya et al. (2019)	Retrospective	153 patients with NSCLC	Hb/RDW ratio, Glasgow prognostic scores, NLR, OS, PFS	Low Hb/RDW was an independent prognostic factor for OS (*p* = 0.03) and PFS (*p* < 0.001) in advanced NSCLC.
[67]	Zhang et al. (2022)	Retrospective	226 patients with breast cancer	Hb/RDW ratio, PLR, monocyte to high-density lipoprotein ratio, risk of breast cancer	Hb/RDW and monocyte to high-density lipoprotein ratio were independent prognostic factors for breast cancer (*p* < 0.001). Low Hb/RDW was linked with prolonged hospitalization, higher RDW, and lower Hb levels (*p* < 0.05).
[68]	Lin et al. (2021)	Retrospective	180 patients with NPC	Hb/RDW ratio, NLR and PLR for the diagnosis of nasopharyngeal cancer	NLR and PLR were notably higher in NPC patients than in healthy subjects (*p* < 0.001). Hb/RDW ratio was extensively lower in NPC patients than in healthy subjects (*p* < 0.001).

Hb: hemoglobin; RDW: red cell distribution width; OS: overall survival; PFS: progression-free survival; DFS: disease-free survival; HR: hazard ratio; ESCC: esophageal squamous-cell carcinoma; RCC: renal cell cancer; LMR: lymphocyte-to-monocyte ratio; NLR: neutrophil-to-lymphocyte ratio; FLOT: fluorouracil, leucovorin, oxaliplatin, docetaxel; DLBCL: diffuse large b-cell lymphoma; EFS: event-free survival; BMI: body mass index; NSCLC: non-small-cell lung cancer; PLR: platelet-to-lymphocyte ratio; NPC: nasopharyngeal cancer.

**Table 2:** GRADE criteria for risk-of-bias evaluation. Green: No Risk of Bias; Yellow: Low or Maybe Risk of Bias; Red: High Risk of Bias.

Reference	Author (Year)	Methodological Quality	Directness of Evidence	Heterogeneity	Precision of Effect Estimates	Publication Bias	Overall Quality of Evidence
[56]	Su et al. (2021)						
[57]	Figen et al. (2023)						
[58]	Petrella et al. (2021)						
[59]	Sun et al. (2016)						
[60]	Yilmaz et al. (2021)						
[61]	Zhao et al. (2022)						
[62]	Yilmaz et al. (2020)						
[63]	Yilmaz et al. (2020)						
[64]	Dong et al. (2022)						
[65]	Tham et al. (2018)						
[66]	Bozkaya et al. (2019)						
[67]	Zhang et al. (2022)						
[68]	Lin et al. (2021)						

## Data Availability

Not applicable.
